# Prevalence of depression and its relationship with quality of life among university students in Macau, Hong Kong and mainland China

**DOI:** 10.1038/s41598-020-72458-w

**Published:** 2020-09-25

**Authors:** Lu Li, Grace K. I. Lok, Song-Li Mei, Xi-Ling Cui, Feng-Rong An, Lin Li, Teris Cheung, Gabor S. Ungvari, Yu-Tao Xiang

**Affiliations:** 1 Department of Pharmacy, The Affiliated Brain Hospital of Guangzhou Medical University, Guangzhou, China; 2 Kiang Wu Nursing College of Macau, Taipa, Macao SAR China; 3 School of Public Health, Jilin University, Changchun, China; 4 Department of Business Administration, Hong Kong Shue Yan University, North Point, Hong Kong SAR China; 5 The National Clinical Research Center for Mental Disorders and Beijing Key Laboratory of Mental Disorders, Beijing Anding Hospital and the Advanced Innovation Center for Human Brain Protection, Capital Medical University, Beijing, China; 6 Department of Pharmacy, The First Affiliated Hospital, College of Medicine, Zhejiang University, Hangzhou, China; 7 School of Nursing, Hong Kong Polytechnic University, Kowloon, Hong Kong SAR China; 8 Division of Psychiatry, School of Medicine, University of Western Australia/Graylands Hospital, Perth, Australia; 9 University of Notre Dame Australia, Fremantle, Australia; 10 Unit of Psychiatry, Institute of Translational Medicine, 3/F, Building E12, Faculty of Health Sciences, University of Macau, Avenida da Universidade, Taipa, Macau SAR China; 11 Center for Cognition and Brain Sciences, University of Macau, Taipa, Macao SAR China

**Keywords:** Neurology, Risk factors

## Abstract

There is compelling evidence that depressive symptoms (depression hereafter) are common in university students and are considerably influenced by the given socioeconomic context. Being former European colonies, Macau and Hong Kong are China’s special administrative regions, with different sociocultural and economic background compared to mainland China. This study compared the prevalence of depression in university students between Macau, Hong Kong and mainland China and examined the association between depression and quality of life (QOL). The Beck Depression Inventory-II and the World Health Organization Quality of Life—Brief Version (WHOQOL-BREF) were used to measure depression and QOL, respectively. Altogether, 2,312 university students participated in this study. The overall prevalence of depression was 28.9%; 35.2% in Macau, 41.0% in Hong Kong, and 16.8% in mainland China. Compared to the “No depression” group, students with depression had significantly lower QOL scores in the physical, psychological, social and environmental domains. Factors associated with depression were different between the three study sites. Sleep disturbances and high academic pressure were positively associated with depression in all the three samples. In mainland China, male students (OR = 1.68; 95% CI: 1.10–2.56) were more likely to have depression while those who were interested in their major (OR = 0.45; 95% CI: 0.29–0.69) were less likely to have depression. In Macau, students in Grade 3 (OR = 0.56; 95% CI: 0.36–0.89) and those who were interested in their major (OR = 0.58; 95% CI: 0.42–0.81) or had optimistic perspective about their future (OR = 0.51; 95% CI: 0.36–0.73) were less likely to have depression. Nursing students (OR = 1.86; 95% CI: 1.21–2.87) and students with the average score on major subject less than 65 (OR = 3.13; 95% CI: 1.70–5.78) were more likely to have depression. In Hong Kong, students with optimistic perspective about their future (OR = 0.44; 95% CI: 0.22–0.91) were less prone to have depression. Depression is common among Chinese university students, particularly in Macau and Hong Kong. Considering the negative impact of depression on QOL, regular screening and effective treatments should be offered to this population.

## Introduction

Depressive symptoms (depression hereafter) are serious health challenge for university students^1,2^. University students are in transition from adolescence to adulthood, and they are confronted by considerable stress in relation to academic demands. Further, they are expected to live independently and maintain good social relationship with others, which probably increase the risk of mental health problems. A meta-analysis found that the global prevalence of depression among baccalaureate students was 30.6%, ranging from 7.9 to 84.5%^3^. The variation of prevalence figures across different areas could be due to the use of different instruments, and a variety of sociocultural and environmental factors^4^.

In 2018, the number of university students in China was 38.33 million accounting for around one fifth of university students globally^5^. In order to reduce the negative health outcomes of depression and develop preventive measures, it is important to understand the epidemiology and risk factors of depression in this segment of the population. Demographic and clinical characteristics significantly associated with depression in university students include female sex^6–8^, family history of depression^4^, and sleep disturbances^9,10^. Good academic results reduced the risk of depression^8,11^. Students who were dissatisfied with their major subject or had learning difficulties were more likely to suffer from depression^8^.

Depressive symptoms such as poor concentration, sadness, loss of interest or pleasure, disturbed sleep or appetite, fatigue, guilty feelings or low self-worth^11^, all have negative impact on quality of life (QOL). QOL is defined as the persons’ perception of their lives in the context of their culture and value systems. QOL is related to the person’s goals, expectations and concerns^12^. QOL comprises the following components of overall health and well-being: physical and mental health, satisfaction with the environment and social relationships^13^.

The epidemiology of mood disorders is considerably influenced by the given socioeconomic context^14–16^, thus the epidemiology of depression in different countries and areas with different socioeconomic backgrounds need to be examined separately. In China, there are special administrative regions which have different sociocultural and political systems, and levels of industrialization and urbanization^17^. Being the former colonies of European countries, the two special administrative regions of China, Hong Kong and Macau, have quite different sociocultural and economic backgrounds compared to the other areas of China. Western upbringing in ways of values, mixed with elements of traditional Chinese culture could increase the risk of depression among young people^18^. To the best of our knowledge, no studies have compared the prevalence of depression in general or in students in particular between Macau, Hong Kong and mainland China. Thus the aim of this study was to examine the prevalence of depression and its relationship with demographic data and QOL in university students in Macau, Hong Kong and mainland China.

## Results

Altogether 2,523 students were invited to participate in this study; 2,312 students completed the study yielding a response rate of 91.6%. The overall prevalence of depression was 28.9% (95% CI: 27.08–30.77%); 35.2% (95% CI: 32.2–38.3%) in Macau, 41.0% (95% CI: 36.56–45.65%) in Hong Kong and 16.8% (95% CI: 14.58–19.37%) in mainland China.

Table 1 shows the basic demographic and clinical characteristics of the whole sample according to study sites. Table 2 presents the comparison between the ‘Depression’ and ‘No-depression’ groups in terms of demographic and clinical characteristics and QOL. After controlling for the potential confounders, students without depression had higher QOL in physical (F_(11, 2312)_ = 315.7, *p* < 0.001), psychological(F_(11, 2312)_ = 474.6, *p* < 0.001), social relationship (F_(11, 2312)_ = 167.3, *p* < 0.001), and environmental (F_(11, 2312)_ = 165.8, *p* < 0.001) domains.

**Table 1:** Socio-demographic characteristics of university students in Macau, Hong Kong and mainland China.

	Macau (n = 928)		Hong Kong (n = 446)		Mainland China (n = 938)		Total sample (n = 2,312)	
	N	%	N	%	N	%	N	%
Male	230	24.8	135	30.3	227	24.2	592	25.6
Religious beliefs	143	15.4	75	16.8	99	10.6	317	14.3
Family history of psychiatric disorders	22	2.4	11	2.5	30	3.2	63	2.7
**Grade**								
First	454	48.9	28	6.3	274	29.2	756	32.7
Second	157	16.9	122	27.4	281	30.0	560	24.2
Third	157	16.9	86	19.3	272	29.0	515	22.3
Fourth or fifth	160	17.2	210	47.1	111	11.8	481	20.8
Nursing students	246	26.5	0	0	232	24.7	478	20.7
**Interested in their major subject**								
Fair or dislike	382	41.2	318	71.3	507	54.1	1,207	52.2
Like	546	58.8	128	28.7	431	45.9	1,105	47.8
**Academic pressure**								
Little or no pressure	647	69.7	358	80.3	700	74.6	1705	73.7
High pressure	281	30.3	88	19.7	238	25.4	607	26.3
**Academic performance**								
85–100	131	14.1	21	4.7	319	34.0	471	20.4
75–84	349	37.6	183	41.0	407	43.4	939	40.6
66–74	287	30.9	180	40.4	154	16.4	621	26.9
< 65	161	17.4	62	13.9	58	6.2	281	12.2
**Perspective on future**								
Fair or pessimistic	502	54.1	390	87.4	553	59.0	1,445	62.5
Optimistic	426	45.9	56	12.6	385	41.0	867	37.5
Sleep disturbances	106	11.4	66	14.8	65	6.9	237	10.3
Depression	327	35.2	183	41.0	158	16.8	668	28.9

	Mean	SD	Mean	SD	Mean	SD	Mean	SD
Age (years)	19.7	1.6	21.2	1.6	20.3	1.4	20.3	1.6
BMI	20.5	3.1	20.1	3.0	21.1	3.4	20.6	3.2

*BMI* body mass index.

**Table 2:** Socio-demographic characteristics of Chinese university students with and without depression.

	No-depression (n = 1644)		Depression (n = 668)		Statistics			Effect size
	N	%	N	%	χ^2^/Z	df^a^	*p*	Cramer’s V
Male gender	405	24.6	187	28.0	2.8	1	0.093	0.035
**Place**					116.4	1	** < 0.001**	0.22
Macau	601	36.6	327	48.9				
Hong Kong	263	16.0	183	27.4				
Mainland China	780	47.4	158	23.7				
Religious beliefs	217	13.2	100	15.0	1.3	1	0.26	0.023
Family history of psychiatric disorders	42	2.6	21	3.1	0.6	1	0.43	0.016
**Grade**					13.5	4	**0.009**	0.076
First	538	32.7	218	32.6				
Second	387	23.5	173	25.9				
Third	396	24.1	119	17.8				
Fourth or fifth	323	19.6	158	23.7				
Nursing students	331	20.1	147	22.0	1.01	1	0.31	0.021
**Interested in their major subject**					44.1	1	**< 0.001**	0.14
Fair or dislike	786	47.8	421	63.0				
Like	858	52.2	247	37.0				
**Academic pressure**					123.6	1	**< 0.001**	0.23
Little or no pressure	1,319	80.2	386	57.8				
High pressure	325	19.8	282	42.2				
**Academic performance**					139.9	4	**< 0.001**	0.26
85–100	395	24.0	76	11.4				
75–84	716	43.6	223	33.4				
66–74	401	24.4	220	32.9				
< 65	132	8.0	149	22.3				
**Perspective on future**					45.9	1	**< 0.001**	0.14
Fair or pessimistic	956	58.2	489	73.2				
Optimistic	688	41.8	179	26.8				
**Sleep disturbances**	95	5.8	142	21.3	123.7	1	**< 0.001**	0.23

	Mean	SD	Mean	SD	T/Z	df^b^	*p*	Cohen’s d
Age (years)	20.2	1.6	20.3	1.6	-0.7	2,310	0.51	0.063
BMI	20.7	3.2	20.5	2.8	1.2	2,310	0.21	0.065
Physical QOL	14.9	2.1	12.6	2.0	24.1	2,310	**< 0.001**	1.11
Psychological QOL	14.4	2.2	11.6	2.2	27.5	2,310	**< 0.001**	1.27
Social QOL	14.0	2.6	12.1	2.5	16.3	2,310	**< 0.001**	0.74
Environmental QOL	13.9	2.3	12.0	2.2	17.9	2,310	**< 0.001**	0.84

Bold values = *p* < 0.05.

*BMI* body mass index, *QOL* quality of life.

^a^chi-square test.

^b^Independent sample T test.

Table 3 shows the independent correlates of depression by study sites. Factors associated with depression were different between the three study sites. Sleep disturbances and high academic pressure were positively associated with depression in all three samples. In mainland China, male students (OR = 1.68; 95% CI: 1.10–2.56) were more likely to have depression while those who were interested in their major (OR = 0.45; 95% CI: 0.29–0.69) were less likely to have depression. In Macau, students in Grade 3 (OR = 0.56; 95% CI: 0.36–0.89) and those who were interested in their major (OR = 0.58; 95% CI: 0.42–0.81) or had optimistic perspective about their future (OR = 0.51; 95% CI: 0.36–0.73) were less likely to have depression. Nursing students (OR = 1.86; 95% CI: 1.21–2.87) and students with the average score on major subject less than 65 (OR = 3.13; 95% CI: 1.70–5.78) were more likely to have depression. In Hong Kong, students with optimistic perspective about their future (OR = 0.44; 95% CI: 0.22–0.91) were less prone to have depression.

**Table 3:** Multiple logistic regression analysis of the correlates of depression by study sites.

Variable	Mainland China			Macau			Hong Kong		
	OR	95% CI	*p*	OR	95% CI	*p*	OR	95% CI	*p*
Male gender	1.68	1.10–2.56	**0.017**	0.88	0.61–1.26	0.48	1.10	0.70–1.72	0.68
Family history of psychiatric disorders	1.15	0.44–3.03	0.78	0.71	0.27–1.87	0.48	1.16	0.31–4.39	0.83
**Grade**									
First	1.0		0.56	1.0		0.007	1.0		0.18
Second	0.75	0.45–1.25	0.27	1.29	0.85–1.96	0.23	2.90	0.99–8.5	0.052
Third	0.70	0.42–1.17	0.18	0.56	0.36–0.89	**0.013**	1.87	0.61–5.73	0.27
Fourth or fifth	0.82	0.43–1.56	0.54	0.70	0.45–1.09	0.12	2.29	0.8–6.53	0.12
**Academic performance**									
85–100	1.0			1.0			1.0		
75–84	0.93	0.59–1.47	0.77	1.13	0.67–1.92	0.65	1.78	0.58–5.50	0.32
66–74	1.04	0.59–1.83	0.89	1.59	0.93–2.74	0.09	2.23	0.71–7.0	0.17
< 65	1.51	0.72–3.15	0.27	3.13	1.70–5.78	**< 0.001**	2.05	0.59–7.05	0.26
Nursing student	0.89	0.54–1.46	0.65	1.86	1.21–2.87	**0.005**	–	–	–
Interested in their major subject	0.45	0.29–0.69	**< 0.001**	0.58	0.42–0.81	**0.001**	1.02	0.62–1.66	0.94
High academic stress	2.98	2.03–4.39	**< 0.001**	2.39	1.71–3.34	**< 0.001**	2.67	1.57–4.53	**< 0.001**
**Perspective on future**									
Fair or pessimistic	1.00			1.00			1.00		
Optimistic	0.71	0.46–1.09	0.12	0.51	0.36–0.73	**< 0.001**	0.44	0.22–0.91	**0.03**
Sleep disturbances	4.00	2.28–7.03	**< 0.001**	2.52	1.61–3.94	**< 0.001**	5.24	2.81–9.74	**< 0.001**

Bold values are *p* < 0.05.

Controlled variables are demographic and clinical variables that significantly associated with depression in univariate analysis and related factors reported association with depression in previous studies (gender, major subject, and family history of psychiatric disorders).

*CI* confidence interval, *OR* odds ratio.

## Discussion

To the best of our knowledge, this was the first study that compared the prevalence of depression in university students between Macau, Hong Kong and mainland China. The prevalence of depression was 28.9% in the whole sample; Hong Kong had the highest prevalence, followed by Macau and mainland China. Post-hoc comparisons with Bonferroni correction found that university students in mainland China were less likely to have depression compared to those in Macau (16.8% vs. 35.2%; χ^2^ = 82.0, *p* < 0.001) and Hong Kong (16.8% vs. 41.0%; χ^2^ = 95.2, *p* < 0.001). There was no significant difference in the prevalence of depression between Macau and Hong Kong at the significance level of 0.017 using Bonferroni corrections. The difference between Hong Kong and mainland China was similar to a previous study (43.9% vs. 24.8%)^19^, while the figure in mainland China was similar to findings of a meta-analysis of 39 studies in Chinese university students (23.8%, 95% CI: 19.9–28.5%)^7^.

Socioeconomic and cultural factors are closely associated with the occurrence and subjective experience of depression^20^. Although the socioeconomic and cultural context characterizing the three sites were not measured due to the lack of standardized instruments in China, we hypothesized that the difference of depression prevalence between the three sites could be partly explained by the interaction and clash between Western lifestyle and traditional Chinese values and culture resulting in the higher rate of depression among Macau and Hong Kong students. Students in Macau and Hong Kong are affected by Western culture and lifestyle although the two cities are Chinese societies. The gap between Western and Chinese traditions may lead to conflicting cultural idioms thereby increasing the risk of depression^19^. Cultural values affect the way people perceive and present with psychological distress, help-seeking behavior and the stigma of mental illness^21,22^. For example, self-control, conformity, and discipline are emphasized in Chinese culture; subthreshold depression, or depressive symptoms are thought to be acceptable to persons growing up with Western values, but in China many people tend to deny depression or only express it with somatic signs and symptoms, which results in lower prevalence of depression^21^. In addition, the higher rate of depression in Macau and Hong Kong could be related to personal characteristics. A comparison between Hong Kong, Shanghai and Beijing students found that Hong Kong students’ self-esteem was significantly lower than those from Shanghai and Beijing, which was associated with higher rate of depression in Hong Kong^23^. Moreover, concealing psychological or psychiatric disturbances among students in mainland China could also result in the lower rate^24^.

Factors associated with depression were different between the three study sites apart from sleep disturbances and high academic pressure that were both positively associated with depression in each site, similar to previous findings^25,26^. A meta-analysis concluded that people with insomnia were more likely to develop depression^27^. Sleep disturbances and depression share risk factors and biological basis; in addition, sleep disturbances are frequent symptoms of depression^8^. Consistent with previous studies^28,29^, students reporting higher academic pressure were more likely to have depression in this study. Ongoing stress could lead to loneliness, fear, helplessness, uselessness, anger, and guilt among other negative feelings and experiences, particularly in individuals with poor coping mechanisms^30^, who are more liable to psychological problems including depression^31,32^. Furthermore, people with low self-esteem^33^ usually have higher level of stress, which is also a contributor to depression.

In this study, an optimistic perspective of the future and greater interest in one’s major subject were negatively associated with depression, which echoes the findings in a study involving Turkish university students^34^. Positive coping styles and attitude reduce the risk of anxiety and depression^35^. Similar to previous findings^8,36,37^, students with average scores of major subject of less than 65 points were more prone to present with depression. Students with poor academic performance cannot meet their parents’ or teachers’ expectations, therefore they experience higher stress, anxiety and low self-esteem, paving the way towards depression^38,39^. Conversely, students with psychiatric disorders, such as depression, are more likely to experience difficulties in their studies^40^.

Compared to other grades, third grade Macau students were less likely to rate themselves depressed, probably because by then they could adapt better to university life while they are not yet looking for jobs and face uncertainty^41^. Unlike in mainland China and Hong Kong, in Macau people graduating elsewhere cannot get a work permit thus Macau students have less pressure related to job-hunting, which may also lower the risk of depression.

Findings regarding gender differences with respect to the prevalence of depression among college students have been contradictory^42,43^. In this study, male students had a higher prevalence of depression in mainland China, but not in Macau and Hong Kong. This may be related to gender differences in seeking psychological help in China, where male students are less likely to utilize mental health services^44^. Inconsistent with results of previous studies^45^, no association was found between family history of mental disorders and depression in either of the sites. Data on family history of mental disorders were collected by self-report in this study, which method is less reliable due to fear of stigma and discrimination. Nursing students were more prone to have depression (OR = 1.86; 95% CI: 1.21–2.87) in Macau, but not in mainland China. We hypothesized that this may be related to the relatively low social status of nursing by the young people. This assumption is supported by the increasingly diminishing rate of applications to nursing schools in Macau.

As expected, depression was positively associated with poor QOL in all domains. Depressed students experience low mood, reduced or loss of interest and pleasure, feelings of guilt or low self-worth, disturbed sleep, low energy, poor concentration, impaired functions, all of which significantly affect their QOL^40^.

The strengths of this study are the large sample size, the inclusion of Macau, Hong Kong and mainland China, and the use of standardized instruments. However, the results should be interpreted with caution due to its methodological limitations. First, this was a cross-sectional study, thus, the causal relationships between depression and other variables could not be investigated. Second, due to logistical reasons, university students were recruited only from Beijing and Jilin province in mainland China. The sample size in Hong Kong was relatively small and only one university in this city participated in the study. The relatively small number of participating universities also limits the generalizability of the findings to all university students in China. In addition, the relatively high percentage of nursing students in the study sample is likely to have caused selection bias. Third, due to logistical reasons, interest in major subject and academic pressure were assessed only by two standardized questions. More sophisticated measures, preferably standardized scales should be used in future studies.

In conclusion, depression was common in Chinese university students, particularly in Macau and Hong Kong. Considering the negative impact of depression on their QOL, regular screening for depressive symptoms should be conducted and effective treatments should be offered to this vulnerable segment of the population.

## Methods

### Study setting and participants

This study was conducted in two nursing colleges (Kiang Wu Nursing College of Macau and Beijing Union Nursing University) and three universities with a number of faculties (University of Macau, Jilin University and Hong Kong ShueYan University) between June and December 2016. In an earlier survey^19^, the prevalence of depression in Hong Kong university students was 43.9%. Given a significance level of 0.05 (two-sided test) and a power of 0.8, at least 436 students were to be recruited in each study site according to the NCSS-PASS program. The University of Macau is the only public university in Macau with around 6,000 undergraduates in eight residential colleges. The Kiang Wu Nursing College of Macau had around 500 nursing students. Jilin University is located in Jilin province with approximately 40,000 undergraduates. Beijing Union Nursing University has around 1,000 undergraduates. Hong Kong Shue Yan University has about 5,000 undergraduates. According to the number of students, grades and residential colleges in participating universities, 2 residential colleges in University of Macau; 4 grades in Kiang Wu Nursing College of Macau; 4 grades in Hong Kong Shue Yan University, 4 grades in Jilin University and 5 grades in Beijing Union Nursing University were randomly selected using computer-generated random numbers. The inclusion criteria were: (1) students aged 18 years and above; (2) fluency in Chinese language (Cantonese or Mandarin); and (3) Willingness to provide written consent and complete the assessment. There were no exclusion criteria. All students in the selected residential colleges and grades were invited to take part in the study on a voluntary basis. Written informed consent was required prior to participation. Students who agreed to participate were given the self-reported questionnaire and were asked to return the completed questionnaires within one week. Participants were assured about anonymity and confidentiality. The study protocol was approved by the Ethics Committees of participating universities.

### Assessment instruments and evaluation

A paper-and-pen version data collection sheet was designed to gather demographic and other information, including gender, age, university, grade, major, level of interest in the major subject, academic pressure, academic performance, and perspective on future.

The presence and severity of depression were measured with the validated Chinese version of the Beck Depression Inventory (BDI-II)^46,47^. The BDI-II consists of 21 items, each ranging from 0 (“symptom not present”) to 3 (“symptom strongly present”). In this study, a total BDI score of 14 or higher indicated depression^47,48^. Sleep disturbances in the previous month were examined with the following standardized questions^49,50^: difficulty initiating sleep (DIS) (“Have you had difficulty falling sleep in the last month?”); difficulty maintaining sleep (DMS) (“Have you had difficulty maintaining sleep or waking up often in the last month?”); early morning awakening (EMA) (“Have you woken up in the middle of the night or early morning in the last month?”). The answers were recorded as “no”, “sometimes” and “often.” The answer of “often” to any of the three questions indicated sleep disturbance^48,51^. Quality of life was evaluated with the World Health Organization Quality of Life—Brief Version (WHOQOL-BREF)^12,19^, which is the most widely used QOL measure in Chinese populations with satisfactory reliability and validity^52,53^. It consists of 26 items covering four domains: physical health, psychological health, and social relationships and environmental factors. A higher score indicates a better QOL.

### Statistical analyses

Data analyses were performed with the SPSS Version 21.0 (Chicago, Ill. USA) for Windows. Normal distribution of continuous variables was measured with Kolmogorov–Smirnov test. Comparisons between students with and without depression in term of socio-demographic and clinical characteristics were made with the independent samples t-test and chi-square test, as appropriate. Multiple chi square tests with Bonferroni correction were conducted to compare the prevalence of depression between Macau, Hong Kong and mainland China. QOL was compared between the ‘Depression’ and ‘No-depression’ groups with analysis of covariance (ANCOVA) after controlling for the potentially confounding effects caused by variables that significantly differed between the two groups in univariate analyses. Multivariate logistic regression analysis using the “Enter” method was performed to determine the independent relationships between depression and the socio-demographic and clinical characteristics. Depression was the dependent variable, while demographic and clinical variables that significantly associated with depression in univariate analysis were the independent variables. In addition, commonly reported associated factors of depression among university students, such as gender^42^, major subject^54^, family history of mental disorders^55^ were entered as the independent variables.

### Human ethics

The study was conducted in accordance with the guidelines of the Declaration of Helsinki. The protocol of the survey has been approved by the Clinical Research Ethics Committee of the University of Macao (Approval number: BSEREAPP002-FHS).

## Data Availability

The data generated and analyzed in this study are available from the corresponding author on request.
